# Unilateral Erosive Arthritis Following Moderna COVID-19 Vaccination

**DOI:** 10.7759/cureus.25020

**Published:** 2022-05-15

**Authors:** Joseph Emran, Sasmith Menakuru, Ibrahim Khan, Vijaypal S Dhillon, Sana Afroz

**Affiliations:** 1 Internal Medicine, Indiana University Health Ball Memorial Hospital, Muncie, USA; 2 Rheumatology, Indiana University Health Ball Memorial Physicians Rheumatology, Muncie, USA

**Keywords:** immunization, vaccination, covid-19, arthritis, erosive arthritis

## Abstract

A novel coronavirus was identified at the end of 2019, causing a pneumonia epidemic in China, which later rapidly spread to cause a global pandemic. However, most people who contracted the COVID-19 had mild to moderate symptoms. A fair percentage developed ARDS, Septic shock, and multi-organ failure. Given the necessity of immunization in combating this disease, COVID-19 vaccines were widely deployed, giving rise to multiple reported cases of post-vaccination autoimmune flareups and new onset of autoimmune phenomena. We present a case of an 81-year-old female who was diagnosed with erosive arthritis post COVID-19 vaccination.

## Introduction

Due to the severity of the COVID-19 pandemic, vaccines were deployed to combat this disease. Post-deployment data showed the effectiveness of the vaccines against COVID-19 risk of hospitalization and death [1]. The majority of COVID-19 vaccine side effects range from mild to moderate local and systemic reactions. However, due to the widespread vaccinations, there is an increase in reported cases of post-COVID-19 vaccine flareup for pre-existing autoimmune diseases and new-onset autoimmune phenomena [2,3]. We present a case of an 81-year-old female who presented with diffuse joint pain and headache, along with elevated inflammatory markers, after receiving the second dose of Moderna COVID-19 vaccine. She was diagnosed with erosive arthritis. The patient was treated with Methotrexate, with symptoms improving on follow-up.

## Case presentation

An 81-year-old female presented as a referral to the outpatient rheumatology clinic due to new complaints of generalized joint pains and a severe headache after receiving the second dose of the Moderna vaccination for SARS-CoV-2. She has a past medical history of osteopenia, thrombophilia on warfarin, and GERD. Her headache was severe, localized to the left temporal area, and not associated with vision changes or jaw claudication. A non-contrast CT head did not show significant findings besides chronic microvascular changes and a left mastoid effusion. Later, a head MRI was done but did not show any significant findings. Given her symptoms, a temporal artery biopsy was performed but did not show any acute or chronic inflammatory changes. Laboratory assessments showed an ESR of 104 mm/hr and a CRP of 4.8 mg/dL. She was referred to a neurologist who started her on a prednisone taper, which she continued for four weeks with minimal improvement in symptoms. Given her history of osteopenia, prednisone was discontinued, and she was referred to rheumatology.

When the rheumatologist saw her, the patient complained of diffuse joint pain. The pain was most pronounced in the right hand, specifically her right third finger, which was also associated with diffuse swelling. The pain was severe to the point of it being debilitating. The patient denied similar symptoms previously. She did not have any history of autoimmune disorders, inflammatory eye disease, inflammatory bowel disease symptoms, or other rheumatological conditions. She also did not have a history of psoriasis, pseudo-gout, and gout. She did not have any rashes or hair loss but did endorse occasional oral ulcerations.

On a focused musculoskeletal examination, bilateral shoulders were tender to palpitation, with reduced active and passive range of motion. Elbows were non-tender with a normal range of motion. The patient had synovitis of the right wrist, second-fourth metacarpophalangeal joint, and right third proximal interphalangeal joint, along with diffuse swelling on the dorsum of the right hand, which was suggestive of tenosynovitis. Her hips were non-tender with a full range of motion bilaterally and mild sacroiliac joint tenderness. Her knees and ankles were also tender bilaterally, with a painful range of motion; however, there was no effusion.

Complete blood count and renal functions were within normal limits. No fluids were analyzed from an affected joint. Another laboratory workup is provided in Table 1.

**Table 1:** Laboratory workup *CRP levels declined after steroid taper **ESR values at one-month interval

Laboratory test	Result	Reference range
ANA antibody	Positive, 1:160 titer, speckled pattern	Normal range <1:80
C3 Complement	106 mg/dL	65 – 180 mg/dL
C4 Complement	26 mg/dL	13 – 52 mg/dL
CRP	Initially 4.8 mg/dL then <0.5 mg/dL*	<=1.0 mg/dL
Anti-dsDNA Ab	Negative	
Anti-SSA Ab	Negative	
Anti-SSB Ab	Negative	
Anti centromere B Ab	Negative	
Anti-chromatin Ab	Negative	
Anti JO 1 Ab	Negative	
Anti Ribosomal P Ab	Negative	
Anti RNP Ab	Negative	
Anti Smith Ab	Negative	
Rheumatoid Factor	Negative	
Anti-CCP	Negative	
Anti SCL70 Ab	Negative	
HLA B27	Negative	
Uric acid	2.2 mg/dL	2.7 – 7.4 mg/dL
ESR	104, 61, 46, 33 mm/hr**	0 – 30 mg/dL

**Figure 1:**
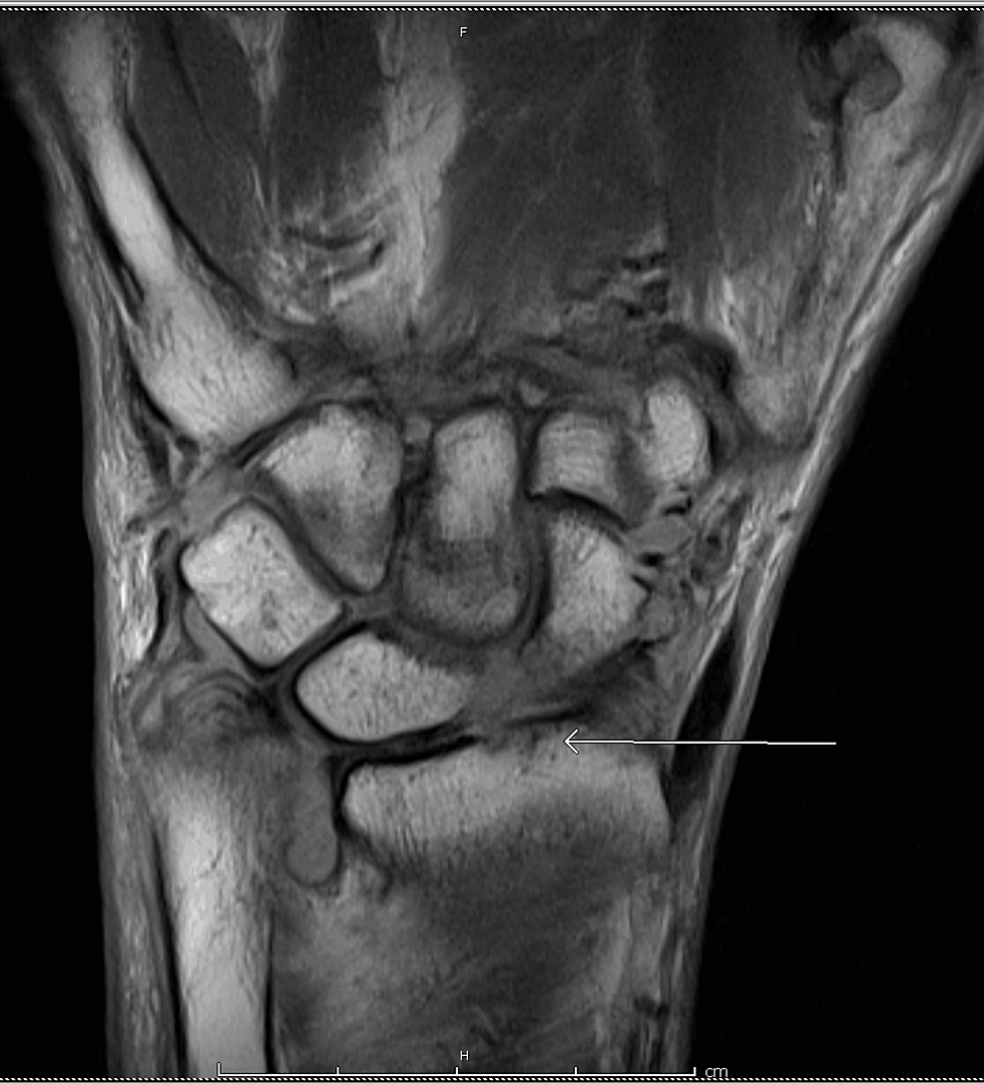
Small erosion at the volar lip of the distal radium

**Figure 2:**
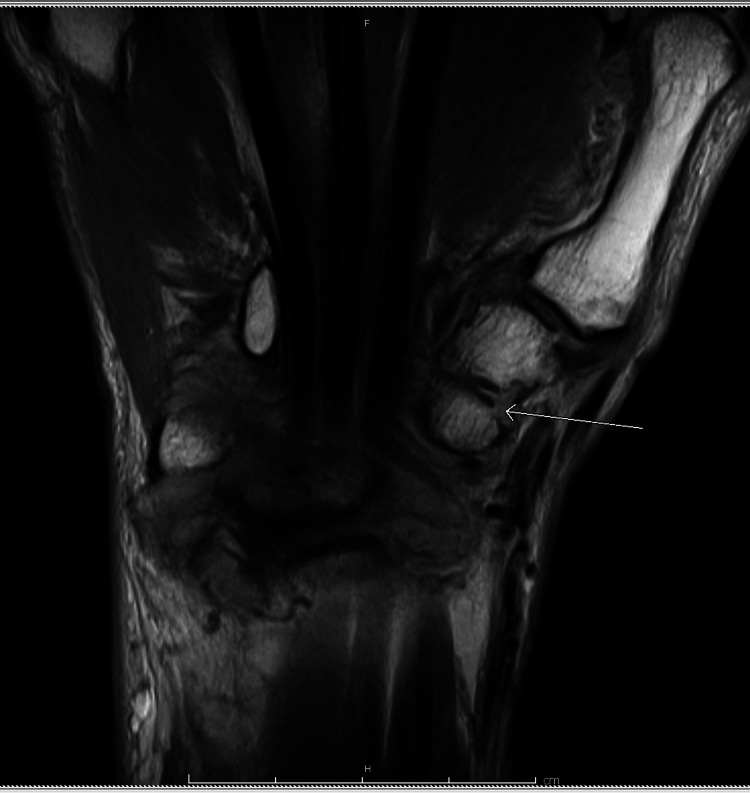
Small erosion at the radial aspect of the trischape joint

**Figure 3:**
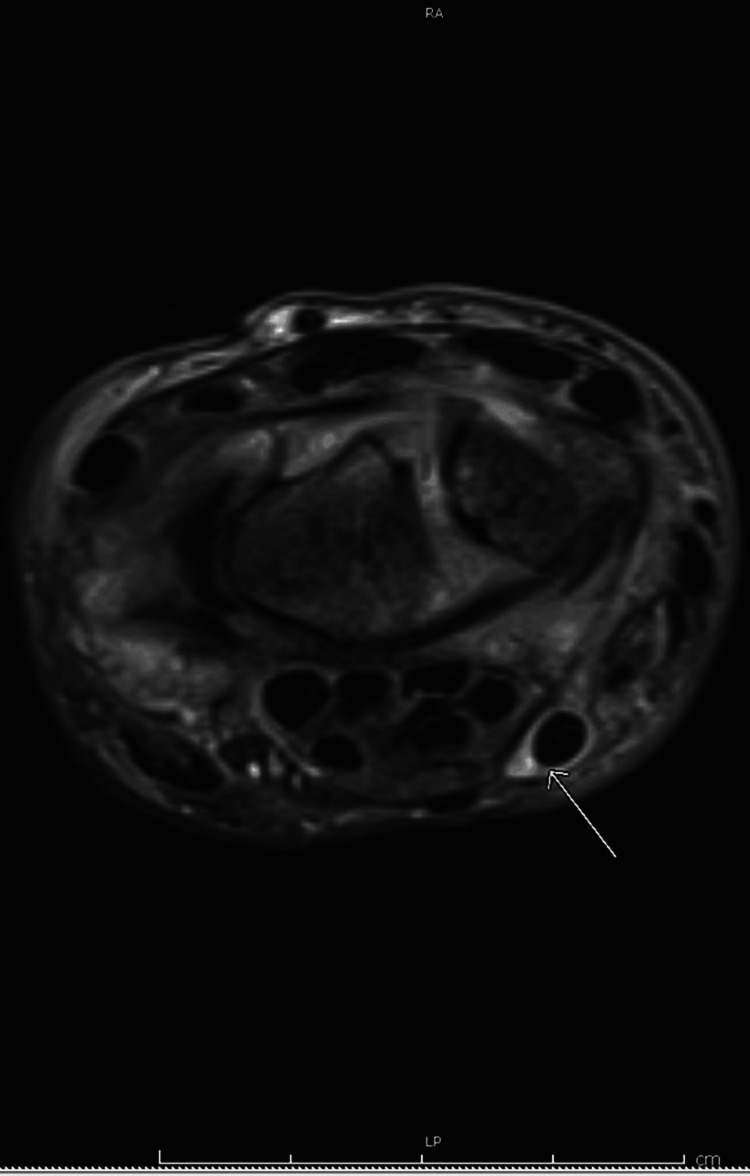
Flexor carpi radialis tenosynovitis

Imaging was done first with a three-view x-ray of her wrists, which showed mild right and left osteoarthritis of the distal interphalangeal joint and the first carpometacarpal joints. X-ray of the bilateral knees showed diffuse osteopenia with minimal degenerative changes. An MRI of the right wrist without contrast showed radiocarpal/intercarpal and distal radioulnar joint synovitis, with scattered intercarpal erosions. There were also findings suggestive of extensor carpi ulnaris and flexor carpi radialis tenosynovitis (Figures 1-3).

The patient was diagnosed with erosive arthritis and seronegative RA. The patient was started on four tablets of 2.5 mg of Methotrexate. A follow-up visit showed improvement in symptoms with residual swelling in the right wrist. A shared decision to go up to six tablets of Methotrexate was made as the patient tolerated the medication well. ESR values at one-month interval progressively declined.

The timeline of symptoms occurred in the following order; the patient had her first Moderna dose in February of 2021, the second dose was in March 2021, and three weeks later, she developed a headache accompanied by multiple joint pain; the joint pain was more pronounced in September of 2021.

## Discussion

The pathogen SARS-CoV-2 emerged late in 2019 and rapidly spread to cause a worldwide pandemic. The clinical course of the disease can range from asymptomatic to severe pneumonia, respiratory failure, septic shock, multi-organ failure, and even death [4]. There has been an increase in reported cases of post-COVID-19 arthritis [5,6]. With the widespread COVID-19 vaccination, there has also been an increase in the number of new-onset autoimmune conditions post COVID-19 vaccination [3].

The post-vaccination inflammatory phenomena have been reported previously with different vaccines, such as the Influenza vaccine (H1N1) and its association with Guillain-Barre syndrome and the HBV vaccine associated with demyelinating neuropathies [7]. The underlying mechanism for such adverse events is assumed to be molecular mimicry. Molecular mimicry refers to a significant similarity between certain pathogenic elements contained in the vaccine and specific human proteins. This similarity may lead to immune cross-reactivity, wherein the reaction of the immune system towards the pathogenic antigens may harm similar human proteins, essentially causing autoimmune disease [7].

Erosive arthritis has a broad differential; in this case, she was diagnosed with seronegative rheumatoid arthritis. This case highlights the importance of recognizing the possibility of post-vaccination inflammatory phenomena. A thorough history, and physical examination, complemented by directed laboratory studies and imaging, can lead to diagnosis and proper management initiation, which can improve the quality of life, help prevent physical impairment down the road, and decrease the financial burden it can pose on the healthcare system if complications arise.

Vaccines are an essential tool in combating various infections. With the widespread promotion of COVID-19 vaccines, we anticipate seeing more autoimmune adverse events. Nonetheless, this should not hinder the vaccine promotion efforts, as the vaccine's benefit outweighs the risks. It is presumed that prone individuals are not only those with previous post-vaccination autoimmune phenomena and those with allergies but also genetically predisposed individuals [8]. Attempts should be made to identify who might be susceptible to developing such reactions and have a patient-centered approach to mitigate these adverse reactions better [3].

It remains unclear whether the COVID-19 vaccine unmasked an underlying autoimmune condition or incited the onset of one. Further investigations of the immunogenic component of the vaccine, as well as the exact underlying mechanism, should be sought, if possible, to better minimize such events from occurring.

## Conclusions

The most plausible mechanism by which the vaccine leads to autoimmune conditions is molecular mimicry, but the exact underlying mechanism is yet to be studied. Given that we do not have a standardized approach to managing post-vaccine autoimmune conditions, management attempts should be tailored according to the diagnosed autoimmune disease. Ongoing immunization against SARS-CoV-2 is encouraged, as the vaccine's benefit outweighs the risk of contracting the virus. Systematic monitoring and ongoing follow-up of autoimmune events will be critical in identifying potential associations between autoimmune manifestations and COVID-19 vaccination, specific mechanisms of diagnosis, and risk stratification for future vaccination.
